# Prognostic value of folate-associated gene expression in stage II colon cancer

**DOI:** 10.1007/s00432-025-06141-w

**Published:** 2025-02-25

**Authors:** Donia Kaidi, Elisabeth Odin, Yvonne Wettergren, Elinor Bexe Lindskog

**Affiliations:** 1 Department of Surgery, Institute of Clinical Sciences, Sahlgrenska Academy, University of Gothenburg, Gothenburg, Sweden; 2 Department of Surgery, Region Västra Götaland, Sahlgrenska University Hospital, Östra, 416 85 Gothenburg, Sweden

**Keywords:** Cancer, Biomarker, Colorectal Neoplams, Prognosis, Folic acid, Thymidylate synthase

## Abstract

**Purpose:**

Prognostic variability in stage II colon cancer underscores the need for better risk stratification. Analyzing folate-associated gene expression in stage II colon cancer could provide researchers and clinicians with deeper insights into tumor biology and potentially aid in identifying early prognostic and/or predictive biomarkers.

**Methods:**

Patients with stage II colon cancer and recurrence (n = 48) were matched to patients with a 5 year recurrence-free follow-up (n = 133). Gene expression of ABCC3, AMT, FPGS, GGH, MFT, PCFT, RFC-1, and TYMS was analyzed in tumor tissue and matching colon mucosa using qPCR and evaluated in relation to time to recurrence (TTR), as well as to demographic and clinicopathological variables.

**Results:**

Independent of other covariates, TYMS expression in tumors, pT4 stage, and emergency surgery were associated with TTR. There were significant differences in expression levels of all examined genes between tumor and mucosa. ABCC3, GGH, and RFC-1 expression levels differed in mucosa between microsatellite instability-high (MSI-H) compared to microsatellite stable/microsatellite instability-low (MSS/MSI-L) tumors, whereas tumoral expression of AMT, GGH, and TYMS differed between MSI-H and MSS/MSI-L tumors. Depending on tumor location, the expression of ABCC3, AMT, GGH, and RFC-1 in mucosa, as well as the tumoral expression of AMT, GGH, PCFT and RFC-1 differed.

**Conclusion:**

Low tumoral expression of TYMS was associated with worse TTR, independent of MSI status, pT stage, and emergency surgery. The indication of a better outcome for patients with MSI-H status and high tumoral TYMS expression might be of particular interest in the stratification of patients for immunotherapy.

**Supplementary Information:**

The online version contains supplementary material available at 10.1007/s00432-025-06141-w.

## Introduction

Colorectal cancer is the third most common cancer globally, with an increasing incidence annually, particularly among patients under 50 years of age (Sinicrope 2022). It is currently one of the leading causes of cancer-related deaths worldwide (Sung et al. 2021). The disease is classified into stages I–IV, with stage I having the best prognosis and stage IV the worst. In stage II colon cancer, the tumor is confined to the colon without lymph node involvement (Nagtegaal et al. 2020). Curative treatment involves surgery alone or surgery combined with chemotherapy, which is considered in the presence of high-risk factors. Examples of high-risk features include tumor perforation during surgery, an insufficient number of examined and removed lymph nodes, poorly differentiated tumors, pT4 stage tumors and perineural invasion (Argilés et al. 2020).

Microsatellite instability (MSI) in tumor DNA, caused by deficient DNA mismatch repair (dMMR), is a well-established positive prognostic factor in stage II colon cancer in terms of relapse-free survival (Argilés et al. 2020). However, since most colon carcinomas are microsatellite stable (MSS), there is a need to identify additional prognostic biomarkers to personalize treatment strategies based on the risk of recurrence for patients with stage II colon cancer. Previous studies by our group have demonstrated that disease-free survival in patients with stage III colorectal cancer differs significantly depending on the expression levels of folate-associated genes (Odin et al. 2015, 2019). Furthermore, there is a known link between folate-associated gene expression and MSI (Odin et al. 2007). However, to our knowledge, there are no studies on MSI status in relation to expression of folate-associated genes in stage II colon cancer.

**Fig. 1:**
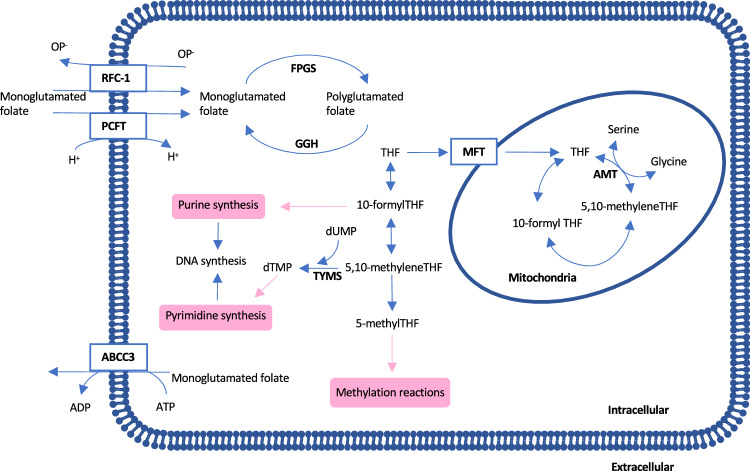
Simplified overview of the folate mediated metabolism, polyglutamation and transport in eukaryotic cells. Polyglutamated folate is converted to monoglutamated folate extracellularly before transport into the cell through the transporter PCFT or RFC-1, depending on pH. MFT transports folates into the mitochondrial matrix. The ATP-dependent protein ABCC3 transports monoglutamated folate amongst other substrates out of the cell. Intracellularly, monoglutamated folate is partly converted into polyglutamated folate by FPGS, whereas GGH hydrolyzes polyglutamated folates to monoglutamated forms. AMT participates in the glycine cleavage system within the mitochondria and is responsible for the formation of 5,10-methyleneTHF. This co-factor forms a ternary complex with dUMP and the TS enzyme, encoded by the *TYMS* gene. Abbreviations: *ATP* Adenosine triphosphate, *dTMP* Deoxythymidine monophosphate, *dUMP* Deoxyuridine monophosphate, *H* Hydrogen, *OP* Organic phosphate, *THF* Tetrahydrofolate

Folate deficiency negatively affects DNA stability, repair, and methylation, increasing the risk of colorectal cancer (Duthie 2011; Giovannucci 2002). Cellular uptake of folates is mediated by folate carriers, transporters, and receptors (Fig. 1). The reduced folate carrier (RFC-1), a member of the solute carrier superfamily, transfers reduced folates, preferably at pH 7.4, whereas the proton-coupled folate transporter (PCFT) operates optimally at pH 5.5 in the gut (Zhao et al. 2009). The mitochondrial folate transporter protein (MFT) transports folates into the mitochondrial matrix. Folate efflux may be mediated by members of the adenosine triphosphate (ATP)-binding cassette (ABC) transporter superfamily, such as ABCC3.The gene ABCC3 encodes an ATP-driven transporter that regulates the outflow of different organic anions, including monoglutamated reduced folates. It belongs to the multidrug resistance protein family because it mediates the efflux of anti-cancer drugs (Kool et al. 1999; Leier et al. 1994).

Within folate metabolism, aminomethyltransferase (AMT), also known as T-protein, participates in the mitochondrial glycine cleavage system. It is responsible for the formation of 5,10-methylenetetrahydrofolate (MeTHF) in the presence of tetrahydrofolate (Fujiwara et al. 1984; Okamura-Ikeda et al. 1987). The TYMS gene encodes the enzyme thymidylate synthase (TS), the target of 5-fluorouracil (5-FU)-based chemotherapy. During cell proliferation, TS forms a ternary complex with 2ʹ-deoxyuridine-5ʹ-monophosphate (dUMP) and MeTHF. When the 5-FU metabolite, FdUMP, replaces dUMP in the this complex, TS is irreversibly inhibited, thereby disrupting the conversion of dUMP to dTMP disrupted.

Intracellular folate homeostasis is regulated by the enzymes folylpolyglutamate synthetase (FPGS) and gamma-glutamyl hydrolase (GGH). To retain a proper folate level and create usable folate derivatives, FPGS links glutamate residues to folate monoglutamates thereby forming polyglutamated folates. The enzyme GGH hydrolyzes polyglutamated folates to monoglutamated forms prior to their export from the cells (Moran 1999).

Analyzing folate-associated gene expression in stage II colon cancer could provide researchers and clinicians with deeper insights into tumor biology and potentially help identify early prognostic and/or predictive biomarkers. The aim of the present study was to investigate whether folate-associated gene expression in tumors and/or mucosa differed between patients with stage II colon cancer who experienced disease recurrence and those who remained recurrence-free, and to determine whether the expression had a prognostic impact on time to recurrence (TTR). Additionally, gene expression was analyzed and evaluated in relation to MSI as well as to demographic and clinicopathological variables, including tumor localization, T stage, and tumor differentiation.

## Patients and methods

### Study population

Patient and tumor characteristics are presented in (Table 1). The patients included in the study were diagnosed between 2002 and 2015. Forty-eight of the patients had radically resected, histologically verified stage II (pT3 or pT4, N0, M0) colon cancer, with available biopsy samples and recurrent disease. These patients were matched according to age and tumor differentiation to patients who were recurrence-free within 5 years of follow up (n = 133). Seven patients received adjuvant chemotherapy due to high-risk features: two in the recurrence group and five in the recurrence-free group. No emergency surgery was performed on these seven patients, four had a pT4 tumor, and four had a high-grade differentiation. Adjuvant chemotherapy was given as 5-fluorouracil (5-FU) in combination with leucovorin to four patients, and as 5-FU/leucovorin plus oxaliplatin to three patients. Exclusion criteria were neoadjuvant intervention, and less than 12 lymph nodes examined. Tumors were classified using the Tumor-Node-Metastasis (TNM) staging system. pT3 tumors were defined as those having grown through the muscularis propria into surrounding pericolorectal tissues without invading nearby organs according to pathological report, while pT4 tumors included those having either penetrated the serosa or invaded adjacent organs or structure according to pathological report (Piñeros et al. 2024). Informed consent was obtained from all patients. The regional ethics committee in Gothenburg, Sweden, approved the study (ethical board number 590-15).

**Table 1:** Patient and tumor characteristics stratified by recurrence in stage II colon cancer patients

	Recurrence (n = 48)	No recurrence (n = 133)	All patients (n = 181)	p value
Age, median (IQR)	74 (64–79.8)	70 (61.5–77)	71 (63–78)	NS
Gender, n (%)				
Female	22 (45.8)	69 (51.9)	91 (50.3)	NS
Male	26 (54.2)	64 (48.1)	90 (49.7)	
Tumor differentiation, n (%)				
Low-grade (G1/G2)	41 (85.4)	109 (84.5)	150 (84.7)	NS
High-grade (G3)	7 (14.6)	20 (15.5)	27 (15.3)	
Tumor location in colon, n (%)				
Right side	22 (45.8)	69 (51.9)	91 (50.3)	NS
Left side	26 (54.2)	64 (48.1)	90 (49.7)	
No. of examined lymph nodes, median (IQR)	20 (14–23.8)	23 (18–27.5)	21 (17–27)	< 0.05
pT stage, n (%)				
pT3	38 (79.2)	125 (94)	163 (90)	< 0.01
pT4	10 (20.8)	8 (6)	18 (10)	
Emergency surgery, n (%)				
No	44 (91.7)	133 (100)	177 (97.8)	< 0.001
Yes	4 (8.3)	0 (0)	4 (2.2)	
Adjuvant chemotherapy, n (%)				
No	46 (95.8)	128 (96.2)	174 (96.1)	NS
Yes	2 (4.2)	5 (3.8)	7 (3.9)	
MSI status, n (%)				
MSI-H	5 (10.4)	34 (25.6)	39 (21.5)	< 0.05
MSI-L/MSS	43 (89.6)	99 (74.4)	142 (78.5)	

*IQR* Interquartile range

^a^Four cases with mucinous tumors were excluded from analysis. The recurrence and no-recurrence groups comprised 48 and 129 cases, respectively

### Preparation of RNA and cDNA

The tumor tissue and matched non-cancerous mucosa (referred to as mucosa throughout the text), obtained approximately 10 cm from the tumor, were snap-frozen in liquid nitrogen and stored at − 80 °C until analysis. Total RNA was isolated from tissue using the AllPrep DNA/RNA Mini Kit (Cat# 80204, Qiagen, Sweden) in accordance with the manufacturer’s instructions. cDNA was synthesized from total RNA using the Applied Biosystems High-Capacity cDNA Reverse Transcription Kit (Cat# 4368814, ThermoFisher Scientific, Sweden) and a T100 Thermal Cycler (Bio-Rad, Sweden).

### Real-time quantitative PCR

The genes selected (Supplementary Table 1) were associated with disease-free survival in previous studies on stage III colorectal cancer (Odin et al. 2015, 2019). PCFT, RFC-1, and ABCC3 are involved in folate transport over the cellular membrane whereas MFT encodes a protein responsible for folate transport into mitochondria. FPGS and GGH have important functions in folate polyglutamation, whereas AMT is involved in mitochondrial folate metabolism. Finally, TYMS was included as it forms a ternary complex with the cofactor MeTHF in both the cytosol and mitochondria. Gene expression was quantified using assays-on-demand (ThermoFisher Scientific, USA) (supplementary Table 1). Samples were analyzed in duplicates. Delta Ct (ΔCt) values were calculated by relating Ct values of target genes to the mean Ct values of the house-keeping genes β-actin (ACTB) and glyceraldehyde-3-phosphate dehydrogenase (GAPDH). The analyses were run on a QuantStudio 12 K Flex qPCR system (Life Technologies, Inc.). The Triplicate Interplate Calibrator (IPC, Cat# IPC250S, TATAA Biocenter AB, Sweden) was used to compensate for interplate variation.

### DNA preparation and microsatellite status

DNA was isolated from snap-frozen tumor and mucosa tissues using the AllPrep DNA/RNA Mini Kit (Cat# 80204 Qiagen, Sweden), or from FFPE tumor tissue using the AllPrep DNA/RNA FFPE kit (Cat# 80234; Qiagen, Sweden). The MSI status was analyzed using the MSI Analysis System, version 1.2 (Promega, Madison, USA), which examined five microsatellites (NR-21, BAT-25, BAT-26, NR-24, and MONO-27). The PCR reaction was run on a T100 Thermal Cycler (Bio-Rad, Sweden) according to the manufacturer’s instructions using two ng of DNA. The MSI markers were detected on an ABI prism 3730 instrument using the PowerPlex 4C matrix Standard (Cat# DG4800, Applied Biosystems, USA). MSI was defined as the peak alterations in the marker electropherogram when tumor tissue was compared with matching mucosa. When more than one of the five markers showed instability, the tumor was defined as MSI high (MSI-H). If only one marker showed instability, it was defined as MSI low (MSI-L). If no instability was detected, the tumor was designated as MSS.

### Statistics

Gene expression data were presented as medians with ranges or as means ± standard deviations (SD). Statistical analyses were performed using GraphPad Prism 10.0.2 software (Boston, MA, USA) and JMP Pro 17.0.0 software (SAS Institute Inc. Cary, NC, USA). Differences between groups were assessed using Pearson’s chi-square test or the Wilcoxon two-sample test. Paired values were analysed using the Wilcoxon matched-pairs signed-rank test. TTR was defined as the time from primary surgery to locoregional recurrence, distant metastases or death from the same cancer. Deaths from other cancers, non-cancer related causes, or treatment-related events were censored, while new colorectal or other cancers were ignored (Birgisson et al. 2011). To estimate the relationship between classical risk factors, gene expression levels in mucosa and tumors, and TTR, univariate Cox proportional hazards regression models were applied to continuous gene expression data, with results presented as hazard ratios (HR) and 95% confidence intervals (CI). Multivariate Cox proportional hazards regression was performed to examine interactions and adjust for potential confounders, including variables found significant in the univariate analysis. The Wald test was used to evaluate the significance of the multivariate model. If one or more genes were found to be significantly associated with TTR in univariate analysis, Kaplan–Meier survival curves were generated to illustrate differences in recurrence-free survival based on gene expression levels. A p value < 0.05 was considered statistically significant. No correction was applied for multiple testing.

## Results

### Gene expression in tumor and mucosa tissue

**Fig. 2:**
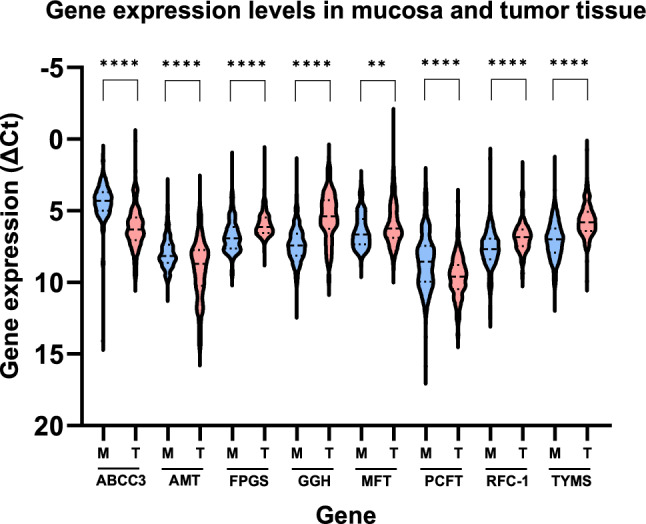
Comparison of gene expression levels in non-cancerous mucosa (M) and tumors (T) obtained at primary surgery from patients with stage II colon cancer. Expression data could be analysed in each of the non-cancerous mucosa samples (n = 181), and in 179/181 tumor samples, except for PCFT (n = 178). Expression levels are presented as violin plots with median values and interquartile ranges depicted as horizontal lines. The scale on the y-axis has been reversed to simplify interpretation as a high ΔCt value represents low gene expression and vice versa. Statistical significance by asterisks, ****p < 0.0001; ***p < 0.001; **p < 0.01; *p < 0.05

Comparison of gene expression in mucosa and tumor tissue is presented in (Fig. 2). As shown, there was a difference in expression of all genes between mucosa and tumors. The expression of ABCC3, AMT and PCFT (p < 0.0001) was higher in mucosa compared to the tumor tissue, whereas the expression of FPGS (p < 0.0001), GGH (p < 0.0001), MFT (p < 0.01), RFC-1 (p < 0.0001) and TYMS (p < 0.0001) was lower in mucosa compared to the tumor tissue.

### Gene expression according to tumor differentiation and pT stage

There was a lower expression of ABCC3 (p < 0.05) in mucosa, and a higher expression of GGH (p < 0.01) in low-grade (G1/G2) compared to high-grade tumors (G3) (supplementary Table 2). The tumoral gene expression of ABCC3 (p < 0.05) was higher in patients with pT4 compared to pT3 tumors (supplementary Table 3).

### Gene expression according to MSI status and tumor location

MSI was detected in 43 of the patients (23.8%) of which four had MSI-L and 39 MSI-H. Thirty-one of the 39 (79.5%) MSI-H tumors were in the right side of the colon. During statistical calculations, the MSS and MSI-L cases were pooled into one group denoted MSS.

**Fig. 3:**
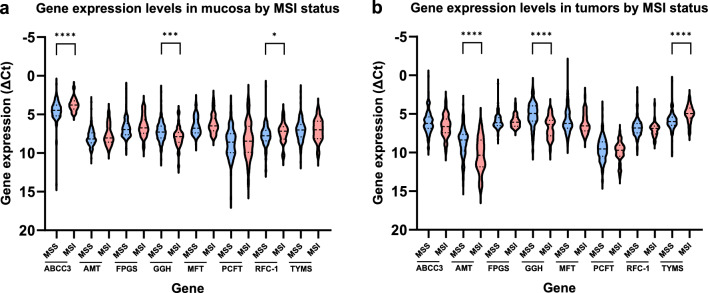
Gene expression levels in **a** non-cancerous mucosa and **b** tumors dichotomized by tumoral MSI status (MSS or MSI). The MSS and MSI-L cases were combined into a single group labelled as MSS, while the MSI-H cases were categorized as the MSI group. Expression levels are presented as violin plots with median values and interquartile ranges depicted as horizontal lines. The scale on the y-axis has been reversed to simplify interpretation as a high ΔCt value represents low gene expression and vice versa. Statistical significance by asterisks, ****p < 0.0001; ***p < 0.001; **p < 0.01; *p < 0.05

Patients with MSI-H tumors had a higher expression of ABCC3 (p < 0.0001), RFC-1 (p < 0.05) and lower gene expression of GGH (p < 0.001) in mucosa compared to patients with MSS/MSI-L tumors (Fig. 3a). They also had a lower gene expression of AMT (p < 0.0001) and GGH (p < 0.0001) and a higher expression of TYMS (p < 0.0001) in tumor tissue (Fig. 3b). The expression of the other genes did not differ between MSI-H and MSS/MSI-L tumors.

**Fig. 4:**
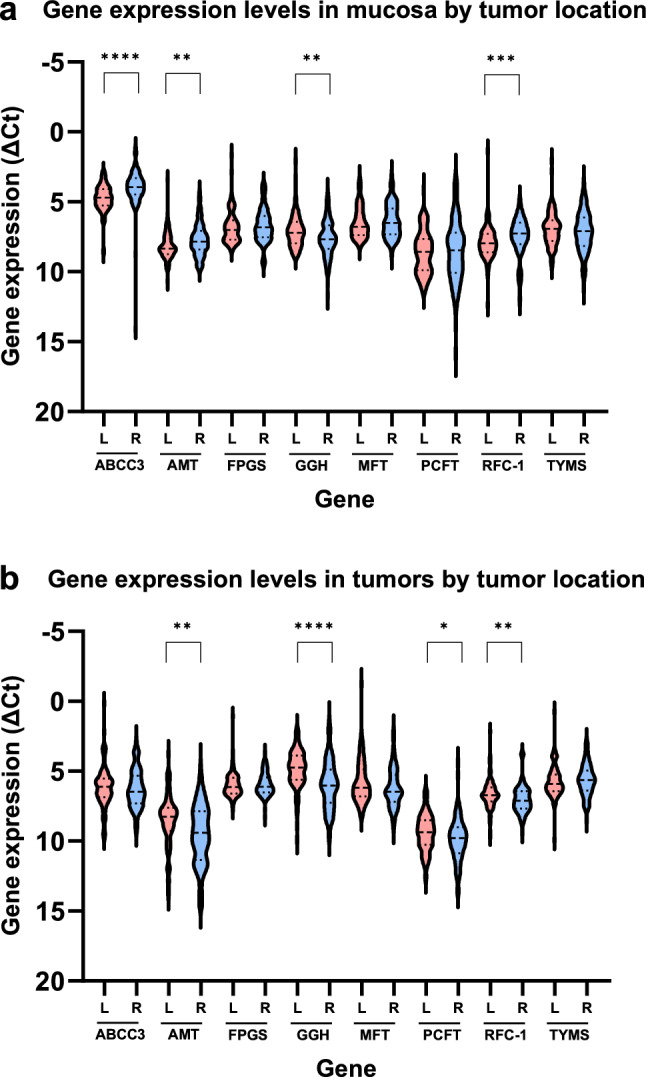
Gene expression levels in **a** non-cancerous mucosa and **b** tumors dichotomized by tumor location (right (R) or left (L) side of colon. Expression levels are presented as violin plots with median values and interquartile ranges depicted as horizontal lines. The scale on the y-axis has been reversed to simplify interpretation as a high ΔCt value represents low gene expression and vice versa. Statistical significance by asterisks, ****p < 0.0001; ***p < 0.001; **p < 0.01; *p < 0.05

Patients with tumors in the right side of colon had higher gene expression of ABCC3 (p < 0.0001), AMT (p < 0.01), and RFC-1 (p < 0.001) and lower gene expression of GGH (p < 0.01) in mucosa, compared to patients with tumors in the left side of colon (Fig. 4a). In tumor tissue of right- compared to left-sided colon cancer, there was a lower gene expression of AMT (p < 0.01), GGH (p < 0.0001), PCFT (p < 0.05), and RFC-1 (p < 0.01) (Fig. 4b).

### Gene expression according to recurrence and prognosis

Patients who experienced recurrence within 5 years of follow-up had a lower expression of TYMS (p < 0.001) in tumor tissue compared to mucosa. Expression of the other genes examined was not different between the recurrence and recurrence-free groups (supplementary Table 4).

**Fig. 5:**
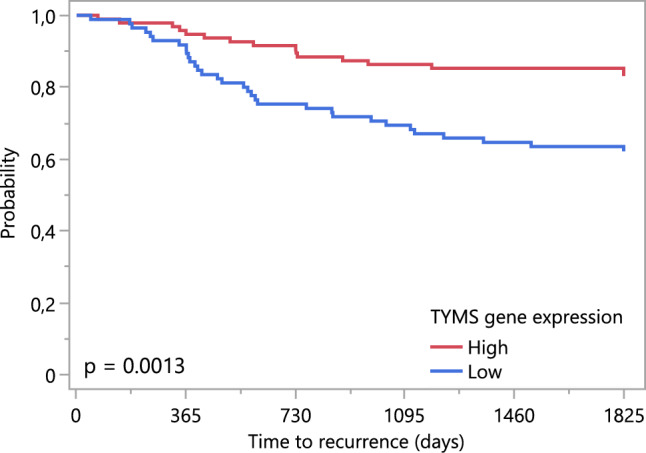
Kaplan–Meier curve showing the probability of time to recurrence (TTR) in stage II colon cancer patients dichotomized by high and low tumoral TYMS gene expression

Univariate Cox regression analysis was performed and showed that low expression of TYMS in tumors was associated with worse TTR. When excluding patients with MSI-H tumors and those who received adjuvant chemotherapy the results remained significant (p < 0.05). To further illustrate the prognostic impact of tumoral TYMS expression, a Kaplan–Meier survival curve was generated, where TYMS expression was dichotomized using the median value 5.8 (Fig. 5). Furthermore, univariate cox regression analysis on clinical and pathological covariates identified an increased risk of recurrence if patients had pT4 compared to pT3 tumors, underwent emergency compared to planned surgery, or had MSI-L/MSS tumors compared to MSI-H (Table 2).

**Table 2:** Cox regression analysis testing the influence of known covariates and the association between gene expression in mucosa and tumor tissue and recurrence-free survival > 5 years in stage II colon cancer patients

	Univariate			Multivariate		
	HR	95% CI	p	HR	95% CI	p
Age	1.02	0.99–1.05	NS			
Gender						
Female	1					
Male	1.23	0.70–2.17	NS			
Tumor differentiation						
Low-grade (G1/G2)	1					
High-grade (G3)	0.91	0.41–2.02	NS			
Tumor location in colon						
Right side	1					
Left side	1.22	0.69–2.16	NS			
No. of examined lymph nodes	0.97	0.93–1.01	NS			
pT stage^a^						
pT3	1			1		
pT4	3.13	1.56–6.30	< 0.01	2.48	1.20–5.13	< 0.05
Emergency surgery						
No	1			1		
Yes	7.1	2.51–20.10	< 0.001	3.68	1.25–10.80	< 0.05
Adjuvant chemotherapy						
No	1					
Yes	1.14	0.28–4.69	NS			
MSI status						
MSI-H	1					
MSI-L/MSS	2.60	1.03–6.57	< 0.05	1.78	0.67–4.69	NS
Gene expression in non-cancerous mucosa						
*ABCC3*	1.15	0.96–1.31	NS			
*AMT*	1.25	0.96–1.66	NS			
*FPGS*	1.12	0.88–1.46	NS			
*GGH*	1.15	0.92–1.43	NS			
*MFT*	1.04	0.82–1.35	NS			
*PCFT*	0.96	0.82–1.11	NS			
*RFC-1*	1.13	0.92–1.39	NS			
*TYMS*	1.06	0.86–1.30	NS			
Gene expression in tumors						
*ABCC3*	0.84	0.70–1.01	NS			
*AMT*	0.91	0.78–1.04	NS			
*FPGS*	1.18	0.84–1.72	NS			
*GGH*	1.12	0.95–1.31	NS			
*MFT*	1.03	0.86–1.26	NS			
*PCFT*	1.00	0.82–1.22	NS			
*RFC-1*	0.94	0.73–1.24	NS			
*TYMS*	1.52	1.19–1.90	< 0.001	1.35	1.04–1.74	< 0.05

Patients were matched by age, and tumor differentiation. Age, gender, tumor differentiation, tumor location in colon, no. of examined lymph nodes, T stage, emergency surgery, adjuvant chemotherapy, MSI status, and gene expression in non-cancerous mucosa (n = 181), gene expression in tumors (n = 179) except for *PCFT* (n = 178). The number of patients included in the multivariate analysis was 179. Excluding patients subjected to adjuvant chemotherapy (n=7), did not affect the significance in the multivariate analysis of the analyzed variables

*ABCC3* ATP-binding cassette, subfamily C (CFTR/MRP), member 3, *AMT* aminomethyltransferase, *FPGS* folylpolyglutamate synthase, *GGH* gamma-glutamyl hydrolase, *MFT* Mitochondrial folate transporter, Solute carrier family 25 (folate transport) member 32, *RFC-1* Reduced folate carrier 1, Solute carrier family 19 (folate transporter), member 1, *PCFT* Proton-coupled folate transporter, Solute carrier family 46 (folate transporter), member 1, *TYMS* Thymidylate synthase, *NS* non-significant

^a^Four cases with mucinous tumors were excluded from analysis

Multivariate Cox regression analysis was performed on the variables that were found to be significant in the univariate analysis. Independent of other covariates, TYMS expression in tumor tissue, pT4 tumors, and emergency surgery were found to be of relevance for TTR. However, MSI-status was not a significant variable in the multivariate analysis (Table 2).

## Discussion

This retrospective case–control study investigated the association between the relative expression of eight folate pathway genes and TTR in 181 patients with stage II colon cancer. The aim was to elucidate whether gene expression in tumor and/or mucosa tissue could serve as a prognostic marker for recurrent disease. The selected genes encode folate transporters (RFC-1, PCFT, ABCC3, and MFT), enzymes involved in the polyglutamation of folates (FPGS and GGH), and the folate metabolism (AMT and TYMS). Low tumoral expression of TYMS, emergency surgery, and pT4 tumors were independently associated with worse TTR. Significant differences in the expression levels of all examined genes were observed between tumors and mucosa, and for some genes, expression also varied with tumor location and MSI status.

Increased expression of RFC-1 and PCFT has been reported during folate deficiency (Thakur et al. 2015), whereas folate over-supplementation leads to downregulation of intestinal folate uptake via decreased transcription of RFC-1 and PCFT (Ashokkumar et al. 2007). The results showed significantly lower RFC-1, but higher PCFT expression in mucosa compared to tumors. High PCFT expression was particularly pronounced in mucosa matching poorly differentiated tumors, suggesting a more acidic microenvironment surrounding these tumors. Additionally, RFC-1 expression was higher in the mucosa of right-sided or MSI-H colon cancers compared to left-sided or MSS/MSI-L cases, whereas the expression of both RFC-1 and PCFT was lower in right- than in left-sided tumors. These findings indicate an association between tumor location (i.e., tumor sidedness) and tissue folate status.

The expression of MFT was higher in tumor tissue compared to mucosa, indicating a high demand for mitochondrial folates in tumors. Although high tumoral expression of MFT has been correlated with reduced survival of patients with different types of solid cancers (Santoro et al. 2020), no association with TTR was found in the present study. The expression of AMT was lower in tumors compared to mucosa, suggesting less AMT activity in the mitochondria of tumor tissue. AMT expression was lower in right-sided or MSI-H colon cancer compared to left-sided or MSS/MSI-L cases, while mucosal AMT expression was higher in right- than left-sided colon cancers, again underscoring the importance of tumor sidedness for certain biological processes.

In agreement with previous studies on chemotherapy-naïve stage I–IV CRC (Hinoshita et al. 2000; Hlavata et al. 2012; Thakur et al. 2015), tumor tissue exhibited significantly lower expression of ABCC3 compared to mucosa. There was no difference in tumoral ABCC3 expression based on tumor location; however, higher expression was observed in the mucosa of right-sided or MSI-H colon cancers compared to left-sided or MSS/MSI-L cases. These results suggest that sidedness-related differences in the efflux of folates or other organic anions in the mucosa may contribute to the development of microenvironments that favor different pathways of tumorigenesis.

In the present study, ABCC3 expression was higher in the mucosa of patients with high-grade (G3) tumors compared to those with low-grade (G1/G2) differentiation. Additionally, ABCC3 expression was elevated in pT4 compared to pT3 tumors. However, ABCC3 expression did not correlate with prognosis nor did it impact TTR. These findings suggest that ABCC3 expression may serve as a potential marker for assessing disease severity and tumor progression, particularly in association with more aggressive disease forms, rather than as a predictor of clinical outcomes such as prognosis.

FPGS catalyzes the polyglutamation of intracellular folates, including MeTHF, which facilitates better stabilization of the ternary complex with the TS enzyme (Chen et al. 2017). In contrast, GGH is involved in removal of folate glutamates in the lysosomes, and it is suggested that monoglutamated folates from the lysosomes are released into the cytosol (Odin et al. 2019). In the present study, both FPGS and GGH were expressed at higher levels in tumors compared to mucosa, indicating an increased intracellular folate turnover in tumors, a finding further supported by the high expression of the proliferation marker TYMS in tumor tissue.

The expression of GGH was lower in both tumor and mucosa of right-sided and MSI-H colon cancers compared to left-sided and MSS/MSI-L cases. A lower expression of GGH was also observed in high-grade (G3) tumors compared to low-grade (G1/G2). This suggests that the availability of polyglutamated folates in the cytosol varies with tumor location and differentiation grade. High FPGS expression has been associated with increased chemosensitivity, whereas GGH overexpression is related to drug resistance (Kim 2020; Schneider and Ryan 2006; Sohn et al. 2004). Therefore, information on FPGS, GGH and TYMS expression may be important when selecting a treatment strategy for high-risk stage II patients who are likely to benefit from adjuvant chemotherapy.

Interestingly, low tumoral expression of TYMS was independently associated with worse TTR, whereas MSI-H correlated with a better TTR, but not independently. In agreement with a previous study, higher TYMS expression was observed in MSI-H tumors, compared to MSS/MSI-L (Odin et al. 2007). MSI-H has been associated with right-sided colorectal cancer and an immune-active tumor microenvironment (Hua et al. 2022), suggesting that high tumor TYMS expression and MSI-H may collectively indicate heightened immune activity within the tumor microenvironment. Contradictory results have been reported regarding the relationship between TYMS expression and prognosis in patients with colorectal cancer (Jensen et al. 2008; Lu et al. 2013; Soong et al. 2008). These discrepant results may have multiple explanations. For example, the TYMS gene is autoregulated; that is, a high transcription does not necessarily lead to increased protein expression and/or TS activity (Tai et al. 2004). Furthermore, differences in study cohorts regarding relevant covariates known to be associated with TYMS expression (e.g., MSI, tumor stages, tumor location) may contribute to these inconsistencies.

### Strengths and limitations

Patients who underwent surgical removal of colon cancer in 2002–2015 were included in this retrospective case–control study. During that time-period, it was not possible to evaluate some of the clinico-pathological parameters which are used today in the assessment of colon cancer, e.g., tumor budding. However, the current study consisted of a relatively large patient cohort which was well-selected and well-controlled. The investigation of the association between MSI status and gene expression gives the study further strength. In the era of next-generation sequencing (NGS), we opted to use qPCR for gene expression analysis. qPCR is particularly well-suited for focused studies targeting specific genes of interest, in contrast to the broad, exploratory scope of NGS. The targeted qPCR methodology offers faster turnaround times compared to NGS, a critical advantage in clinical settings where timely decision-making is essential. It also provides high sensitivity and specificity, enabling the detection of low-abundance transcripts and subtle changes in gene expression. The study examined the relative expression of the selected genes, but not the corresponding protein expression or enzyme activities. In future studies, it will be valuable to analyze the correlation between TYMS gene and protein expression in tumor tissues and to investigate the possible association between TS activity and TTR. It will also be of interest to evaluate folate-associated gene expression and MSI status in relation to tumor budding and other relevant parameters. To address these questions, a follow-up study of the patient cohort is planned.

## Conclusions

There were significant differences in expression levels of genes involved in folate transport, metabolism and polyglutamation between tumor and matched non-cancerous mucosal tissue. Significant differences in gene expression related to tumor location and/or MSI status were seen in both mucosa and tumor tissues. The effects of these differences on tumorigenesis are currently unknown, highlighting the need for further investigation of the interaction between the tumor and its surrounding mucosa in terms of folates and other metabolites. Low tumoral TYMS expression was associated with a worse TTR, independent of MSI status, pT stage and emergency surgery. Thus, TYMS may have prognostic value for patients with stage II colon cancer. To fully understand whether TYMS gene expression is clinically important in early colon cancer, extended studies evaluating its correlation with protein expression and TS enzyme activity are needed. Additionally, the indication of a better outcome for patients with MSI-high status and high tumoral TYMS expression could be particularly relevant when selecting patients for immunotherapy.

## Supplementary Information

Below is the link to the electronic supplementary material.

Supplementary file1 (DOCX 38 KB)

## Data Availability

No datasets were generated or analysed during the current study.
