# Supplementary material for: Prognostic value of folate-associated gene expression in stage II colon cancer
Source: J Cancer Res Clin Oncol. 2025 Feb 25;151(2):92. doi: 10.1007/s00432-025-06141-w (PMC11861115; doi:10.1007/s00432-025-06141-w)
Supplement: Supplementary file 1 — Supplementary file1 (DOCX 38 KB) [file 432_2025_6141_MOESM1_ESM.docx]

| **Supplementary Table 1**. List and assay ID of analyzed genes | | | |
| --- | --- | --- | --- |
| Gene category | Gene | Gene name | Assay ID |
| **Folate transport** |  |  |  |
|  | *ABCC3* | ATP-binding cassette, subfamily C member 3; MRP3: Multidrug resistance-associated protein 3 | Hs00358656_ml |
|  | *RFC-1* | Reduced folate carrier 1; SLC19A1: Solute carrier family 19 (folate transporter), member 1 | Hs00953344_ml |
|  | *PCFT* | Proton-coupled folate transporter; SLC46A1: Solute carrier family 46 (folate transporter), member 1 | Hs00611081_m1 |
|  | *MFT* | Mitochondrial folate transporter; SLC25A32: Solute carrier family 25 (folate transport) member 32 |  |
| **Folate metabolism** |  |  |  |
|  | *AMT* | Aminomethyltransferase; T-protein | Hs00166628_m1 |
|  | *TYMS* | Thymidylate synthase |  |
| **Folate polyglutamation** |  |  |  |
|  | *FPGS* | Folylpolyglutamate synthase | Hs00191956_m1 |
|  | *GGH* | γ-Glutamyl hydrolase; conjugase, folylpolygammaglutamyl hydrolase | Hs00914163_m1 |
| **House-keeping** |  |  |  |
|  | *ACTB* | β-actin | Hs99999903_m1 |
|  | *GAPDH* | Glyceraldehyde-3-phosphate dehydrogenase | Hs99999905_m1 |
| **Abbreviations:** ATP, adenosine triphosphate. | | | |

# Prognostic value of folate-associated gene expression in stage II colon cancer

Journal of Cancer Research and Clinical Oncology
Donia Kaidi, Elisabeth Odin, Yvonne Wettergren and Elinor Bexe Lindskog

*Corresponding author:*
Elinor Bexe Lindskog, ORCID 0000-0003-1466-1486

Department of Surgery, Institute of Clinical Sciences, Sahlgrenska Academy, University of Gothenburg, Gothenburg, Sweden. Region Västra Götaland, Sahlgrenska University Hospital, dept of Surgery, Gothenburg, Sweden.
[elinor.bexe-lindskog@surgery.gu.se](mailto:elinor.bexe-lindskog@surgery.gu.se)

| **Supplementary table 2**. Gene expression levels in mucosal and tumor tissues stratified by tumor differentiation in stage II colon cancer patients | | | | |
| --- | --- | --- | --- | --- |
| Gene | Gene category | Gene expression (ΔC_t_) | | *p*-Value |
|  |  | Low-grade differentiation (G1/G2) | High-grade differentiation (G3) |  |
| **Non-cancerous mucosa tissue^a^** |  |  |  |  |
| *ABCC3* | Transport | 4.44 ± 1.38 | 3.92 ± 0.69 | <0.05 |
| *AMT* | Metabolism | 7.97 ± 1.09 | 7.62 ± 1.21 | NS |
| *FPGS* | Polyglutamation | 6.70 ± 1.17 | 6.93 ± 1.99 | NS |
| *GGH* | Polyglutamation | 7.31 ± 1.28 | 7.32 ± 1.09 | NS |
| *MFT* | Transport | 6.38 ± 1.16 | 6.66 ± 1.21 | NS |
| *PCFT* | Transport | 8.60 ± 1.77 | 7.95 ± 1.99 | NS |
| *RFC-1* | Transport | 7.71 ± 1.35 | 7.44 ± 1.13 | NS |
| *TYMS* | Metabolism | 7.01 ± 1.34 | 7.04 ± 1.38 | NS |
| **Tumor tissue^b^** |  |  |  |  |
| *ABCC3* | Transport | 6.11 ± 1.33 | 6.46 ± 1.84 | NS |
| *AMT* | Metabolism | 8.88 ± 2.00 | 9.65 ± 2.37 | NS |
| *FPGS* | Polyglutamation | 5.95 ± 0.82 | 5.99 ± 1.09 | NS |
| *GGH* | Polyglutamation | 5.27 ± 1.64 | 6.17 ± 1.66 | <0.01 |
| *MFT* | Transport | 5.97 ± 1.45 | 5.85 ± 1.63 | NS |
| *PCFT* | Transport | 9.57 ± 1.49 | 9.86 ± 1.21 | NS |
| *RFC-1* | Transport | 6.85 ± 1.02 | 6.76 ± 1.18 | NS |
| *TYMS* | Metabolism | 5.78 ± 1.10 | 5.32 ± 1.27 | <0.05 |
| **Abbreviations:**  *ABCC3*: ATP-binding cassette, subfamily C (CFTR/MRP), member 3; *AMT*: aminomethyltransferase; *FPGS*: folylpolyglutamate synthase; *GGH*: gamma-glutamyl hydrolase; *MFT*: Mitochondrial folate transporter, Solute carrier family 25 (folate transport) member 32; *RFC-1*: Reduced folate carrier 1, Solute carrier family 19 (folate transporter), member 1; *PCFT*: Proton-coupled folate transporter, Solute carrier family 46 (folate transporter), member 1; *TYMS*: Thymidylate synthase. **Notes:** Four cases with mucinous tumors were excluded from the analysis. ^a^Low grade (n=150), high-grade (n=27), ^b^Low grade (n=148) except for *PCFT* (n**=**147**),** high-grade (n=27). Values are calculated using the ΔC_t_ method (i.e., high ΔC_t_ values correspond to low gene expression levels) and expressed as mean ± SD. NS: non-significant. | | | | |

| **Supplementary Table 3**. Gene expression levels in mucosa and tumor tissue stratified by pT stage in stage II colon cancer patients | | | | |
| --- | --- | --- | --- | --- |
| Gene | Gene category | Gene expression (ΔC_t_) | | *p*-Value |
|  |  | pT3 | pT4 |  |
| **Non-cancerous mucosa tissue^a^** |  |  |  |  |
| *ABCC3* | Transport | 4.31 ± 1.09 | 4.95 ± 2.49 | NS |
| *AMT* | Metabolism | 7.92 ± 1.13 | 8.07 ± 0.92 | NS |
| *FPGS* | Polyglutamation | 6.76 ± 1.22 | 6.69 ± 0.78 | NS |
| *GGH* | Polyglutamation | 7.30 ± 1.29 | 7.88 ± 1.23 | NS |
| *MFT* | Transport | 6.44 ± 1.19 | 6.38 ± 0.98 | NS |
| *PCFT* | Transport | 8.58 ± 1.93 | 8.59 ± 1.87 | NS |
| *RFC-1* | Transport | 7.72 ± 1.37 | 7.57 ± 0.94 | NS |
| *TYMS* | Metabolism | 7.07 ± 1.36 | 6.83 ± 1.35 | NS |
| **Tumor tissue^b^** |  |  |  |  |
| *ABCC3* | Transport | 6.25 ± 1.36 | 5.63 ± 1.88 | <0.05 |
| *AMT* | Metabolism | 9.09 ± 2.08 | 8.41 ± 1.77 | NS |
| *FPGS* | Polyglutamation | 5.95 ± 0.82 | 6.04 ± 1.12 | NS |
| *GGH* | Polyglutamation | 5.38 ± 1.68 | 5.75 ± 1.54 | NS |
| *MFT* | Transport | 6.01 ± 1.44 | 5.74 ± 1.79 | NS |
| *PCFT* | Transport | 9.65 ± 1.46 | 9.61 ± 1.53 | NS |
| *RFC-1* | Transport | 6.89 ± 0.96 | 6.36 ± 1.51 | NS |
| *TYMS* | Metabolism | 5.67 ± 1.10 | 5.89 ± 1.40 | NS |
| **Abbreviations**: *ABCC3*: ATP-binding cassette, subfamily C (CFTR/MRP), member 3; *AMT*: aminomethyltransferase; *FPGS*: folylpolyglutamate synthase; *GGH*: gamma-glutamyl hydrolase; *MFT*: Mitochondrial folate transporter, Solute carrier family 25 (folate transport) member 32; *RFC-1*: Reduced folate carrier 1, Solute carrier family 19 (folate transporter), member 1; *PCFT*: Proton-coupled folate transporter, Solute carrier family 46 (folate transporter), member 1; *TYMS*: Thymidylate synthase. **Notes:**  ^a^pT3 (n=163), pT4 (n=18),  ^b^pT3 (n=161), pT4 (n=18) except for *PCFT* pT4 (n=17). Values are calculated using the ΔC_t_ method (i.e., high ΔC_t_ values correspond to low gene expression levels) and expressed as mean ± SD. NS: non-significant. | | | | |

| **Supplementary table 4**. Gene expression levels in mucosa and tumor tissue stratified by recurrence in stage II colon cancer patients | | | | |
| --- | --- | --- | --- | --- |
| Gene | Gene category | Gene expression (ΔC_t_) | | *p*-Value |
|  |  | Recurrence | Recurrence-free |  |
| **Non-cancerous mucosa tissue^a^** |  |  |  |  |
| ABCC3 | Transport | 4.65 ± 1.23 | 4.27 ± 1.31 | NS |
| AMT | Metabolism | 8.18 ± 0.82 | 7.85 ± 1.18 | NS |
| FPGS | Polyglutamation | 6.90 ± 1.00 | 6.70 ± 1.24 | NS |
| GGH | Polyglutamation | 7.55 ± 1.08 | 7.29 ± 1.36 | NS |
| MFT | Transport | 6.50 ± 1.04 | 6.41 ± 1.22 | NS |
| PCFT | Transport | 8.46 ± 1.56 | 8.62 ± 2.04 | NS |
| RFC-1 | Transport | 7.91 ± 1.16 | 7.63 ± 1.39 | NS |
| TYMS | Metabolism | 7.15 ± 1.24 | 7.01 ± 1.40 | NS |
| **Tumor tissue^b^** |  |  |  |  |
| ABCC3 | Transport | 5.88 ± 1.42 | 6.30 ± 1.42 | NS |
| AMT | Metabolism | 8.72 ± 2.01 | 9.14 ± 2.07 | NS |
| FPGS | Polyglutamation | 6.07 ± 0.75 | 5.92 ± 0.89 | NS |
| GGH | Polyglutamation | 5.68 ± 1.49 | 5.32 ± 1.72 | NS |
| MFT | Transport | 6.04 ± 1.15 | 5.96 ± 1.58 | NS |
| PCFT | Transport | 9.64 ± 1.41 | 9.64 ± 1.48 | NS |
| RFC-1 | Transport | 6.78 ± 0.94 | 6.86 ± 1.07 | NS |
| TYMS | Metabolism | 6.15 ± 0.77 | 5.52 ± 1.20 | <0.001 |
| **Abbreviations:** ABCC3: ATP-binding cassette, subfamily C (CFTR/MRP), member 3; AMT: aminomethyltransferase; FPGS: folylpolyglutamate synthase; GGH: gamma-glutamyl hydrolase; MFT: Mitochondrial folate transporter, Solute carrier family 25 (folate transport) member 32; RFC-1: Reduced folate carrier 1, Solute carrier family 19 (folate transporter), member 1; PCFT: Proton-coupled folate transporter, Solute carrier family 46 (folate transporter), member 1; TYMS: Thymidylate synthase. **Notes:** ^a^Recurrence (n=48), Recurrence-free (n=133), ^b^Recurrence (n=48), Recurrence-free (n=131), except for PCFT Relapse (n=47). Values are calculated using the ΔC_t_ method (i.e., high ΔC_t_ values correspond to low gene expression levels) and expressed as mean ± SD. NS: non-significant. | | | | |
